# Multifocal Disseminated Methicillin‐Sensitive *Staphylococcus aureus* Bacteremia With Complex Multiorgan Involvement: A Case Report

**DOI:** 10.1155/crdi/6645912

**Published:** 2026-01-30

**Authors:** Cameron Vicknair, Linda Akbarshahi, Tahani Dakkak, Leslie David

**Affiliations:** ^1^ Department of Graduate Medical Education, Northeast Georgia Medical Center, Gainesville, Georgia, USA; ^2^ Department of Family Medicine, Northeast Georgia Medical Center, Gainesville, Georgia, USA

## Abstract

**Background:**

Methicillin‐sensitive *Staphylococcus aureus* (MSSA) bacteremia is a bloodstream infection that can lead to a wide range of complications, from localized skin infections to serious and systemic conditions. While MSSA remains susceptible to beta‐lactam antibiotics, its potential for hematogenous dissemination poses significant clinical challenges impacting multiple organ systems and causing substantial morbidity.

**Clinical Presentation:**

We present the case of a 61‐year‐old male patient who developed disseminated MSSA bacteremia following suspected soft tissue inoculation events. His clinical course was complicated by spinal epidural abscess, right atrial thrombus, chest wall abscess, septic arthritis, prevertebral abscess, left psoas abscess, and gluteal abscess. He underwent T6 laminectomy with evacuation of the epidural abscess, debridement of the chest wall abscess, percutaneous thromboembolectomy, and multiple incisions and drainage procedures. A multidisciplinary approach was essential in achieving clinical improvement.

**Conclusion:**

This case underscores the aggressive potential of disseminated MSSA bacteremia to spread beyond its initial source, resulting in widespread organ involvement. Early recognition through imaging, prompt source control, and targeted antimicrobial therapy are critical in managing the complex sequelae of this condition.

## 1. Introduction

Methicillin‐sensitive *Staphylococcus aureus* (MSSA) is a common pathogen implicated in a broad spectrum of clinical diseases. *S. aureus* colonizes the skin and mucous membranes in approximately 30% of the population without causing harm [1]. However, when the bacterium breaches the skin or mucosal barrier and enters the bloodstream, it may predispose individuals to severe infections, including bacteremia [2]. Population studies show that the incidence rate for MSSA‐related bacteremia ranges from 20 to 50 cases/100,000, with 20%–30% of these cases resulting in mortality [3]. Several risk factors predispose individuals to MSSA bacteremia, including advanced age, injection drug use, or the presence of intravascular catheters and prosthetic devices [4]. Additionally, underlying medical conditions such as diabetes, renal disease dependent on dialysis, rheumatoid arthritis, malignancy history, transplant history, and HIV [5] also increase susceptibility to MSSA bacteremia.

While *S. aureus* is a leading cause of bacteremia [6], the pathogens’ metastasis is rarer and more unpredictable, with incidences between 13% and 39% [7–13]. Studies have shown that MSSA can invade distant anatomical sites and cause serious metastatic infections, including endocarditis, septic arthritis, vertebral osteomyelitis, spinal epidural abscess, and psoas abscess [14]. Although MSSA is susceptible to beta‐lactam antibiotics, it remains a significant cause of morbidity when complicated by hematogenous spread to multiple organ systems. This case highlights the diagnostic and therapeutic complexity of managing disseminated MSSA bacteremia with multiorgan involvement.

## 2. Case Presentation

A 61‐year‐old male with a history of cervical disc disease, bipolar disorder, recurrent depression, peripheral neuropathy, cerebrospinal fluid leak status, posttraumatic brain injury, and prior cervical spine injury was evaluated.

In late 2023, the patient experienced progressive back pain, fatigue, and weight loss, raising suspicion of spinal infection. The patient had a prior history of C5–C7 fusions. He was also previously diagnosed with nondiabetic peripheral neuropathy attributed to chronic C5/C6 radiculopathy, multilevel degenerative cervical spine disease, and carpal tunnel syndrome, as confirmed by electromyography. Imaging at the time showed mild disc space narrowing and degenerative changes, though no definitive infection. Additionally, there were no abscesses or overt signs of osteomyelitis. Laboratory values demonstrated no elevation in erythrocyte sedimentation rate (ESR) or C‐reactive protein (CRP) inflammatory marker levels at this time. Conservative treatment was initiated but failed to relieve symptoms.

Throughout 2024, the patient continued to report intermittent exacerbations of back and joint pain, particularly in the left hip, knee, and neck. Several urgent care and emergency visits noted joint tenderness and nonspecific laboratory abnormalities. The patient was prescribed intramuscular (IM) testosterone, which he self‐administered. In mid‐2024, he sustained a hand injury when a wrench fell on the dorsum of the hand. These two intermittent inoculation events are now considered likely entry points for hematogenous seeding by *S. aureus*, as chronic *S. aureus* latency is unlikely. Although there is no direct clinical evidence that definitively links the hand injury to the upper limb injury, given the timing and mechanism of the injuries, both events were considered plausible portals for the same inoculation pathway that led to infection.

**FIGURE 1:**
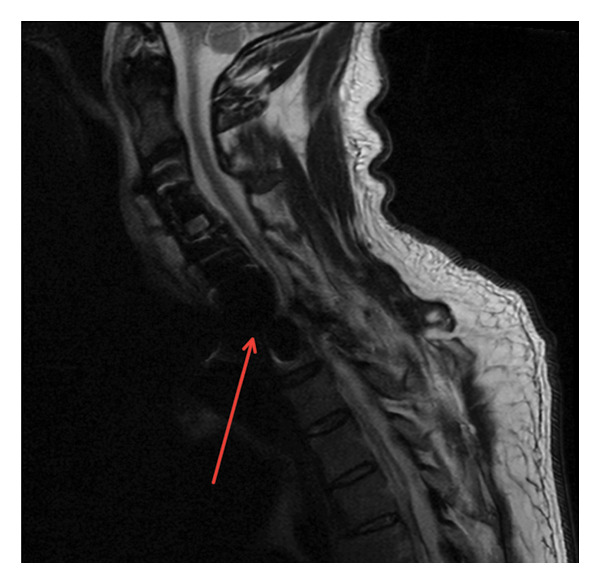
Cervical and thoracic spine MRI showing a partially imaged, extensive posterior epidural collection consistent with abscess formation extending from C7 through the thoracic spine (red arrow). Additional findings include prevertebral phlegmon and suspected abscess from C3 to C5, as well as contrast enhancement adjacent to the prior C3–C5 anterior cervical graft, suggestive of granulation tissue or infectious/inflammatory changes. Multilevel spondylotic changes are also noted.

In April 2025, the patient presented with severe back pain. MRI revealed a dorsal spinal abscess from T2 to T6, with severe central canal stenosis and cord compression, along with signs of osteodiscitis and a left psoas abscess. MRI of the cervical and thoracic spine confirmed multilevel involvement with adjacent soft tissue abscess concerning hematogenous MSSA dissemination (Figure 1).

On May 1, 2025, the patient underwent T6 laminectomy with evacuation of the epidural abscess. Intraoperative cultures from the thoracic spine revealed numerous white blood cells (WBCs) and Gram‐positive cocci in clusters consistent with *S*. *aureus*. Blood cultures confirmed MSSA (Table 1). The patient was initiated on cefazolin 2 g IV every 6 h.

**TABLE 1:** Laboratory investigations.

Test	Result	Reference range	Interpretation
WBC	16.6	4.0–11.0	High
HGB	9.2	13.5–17.5	Low
HCT	28.5	38%–50%	Low
MCV	91	80–100 fL	Normal
PLT	410	150–400	Slightly high
Na	135	136–145	Low normal
K	4.2	3.5–5.1	Normal
Cl	100	98–107	Normal
CO_2_	24	22–30	Normal
BUN	18	7–20	Normal
Cr	0.88	0.7–1.3	Normal
Glucose	110	70–99	High
Ca	9.0	8.5–10.5	Normal
Mg	2.1	1.7–2.3	Normal
AST	38	10–35	High
ALT	35	10–40	Normal
Alk Phos	102	44–147	Normal
Total Bili	0.5	0.1–1.2	Normal
Procalcitonin	3.2	< 0.15	Elevated
CRP	118	< 3.0	Elevated
Blood Culture	MSSA Positive	Negative	Abnormal

*Note:* HGB, hemoglobin; HCT, hematocrit; PLT, platelet count; Na, sodium; K, potassium; Cl, chloride; CO_2_, bicarbonate; Cr, creatinine; Ca, calcium; Mg, magnesium; AST, aspartate aminotransferase; ALT, alanine aminotransferase; Alk Phos, alkaline phosphatase; CRP, C‐reactive protein; Total Bili, total bilirubin; WBC, white blood cell count.

Abbreviations: BUN, blood urea nitrogen; MCV, mean corpuscular volume.

The following day, he underwent debridement of a right pectoralis/chest wall abscess and incision and drainage of a hand abscess. A repeat blood culture on May 3 grew MSSA. The following day, a second‐look debridement of the chest wall wound was performed with wound vacuum placement. The patient reported left knee pain with tingling sensations the next day. Arthrocentesis was performed, yielding few WBCs, no organisms, and calcium pyrophosphate crystals consistent with pseudogout.

On May 6, the patient developed hoarseness and neck swelling. CT imaging revealed a prevertebral abscess from C2–C7. An incision and drainage of the C2–C7 prevertebral abscess was performed, revealing WBCs, few Gram‐positive cocci, and no anaerobic organisms. Bilateral knee incision and drainages were performed due to worsening swelling and concern for septic arthritis; cultures were negative.

**FIGURE 2:**
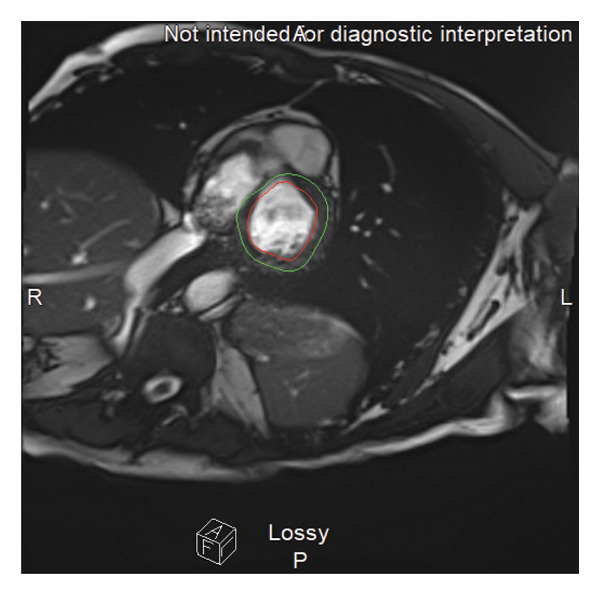
MRI of the heart demonstrating a mobile right atrial mass, intermittently visualized, appearing to arise from the Eustachian valve near the inferior vena cava (red circle). The mass is not attached to any cardiac valves, and there is no associated valvular pathology. These findings are more consistent with thrombus rather than infective vegetation.

On May 13, a transesophageal echocardiogram (TEE) revealed a large, multilobular, mobile mass in the right atrium with a positive bubble study indicating intracardiac shunt. MRI findings favored thrombus over vegetation (Figure 2). Valves were intact on both TEE and MRI. Three days after the TEE, the patient underwent a percutaneous suction thromboembolectomy, retrieving a 2.5‐cm right atrial mass. Cultures revealed few WBCs, no organisms, and negative anaerobic and fungal cultures. The patient developed persistent sinus tachycardia; computed tomography angiography revealed ground‐glass opacities bilaterally and mild hepatomegaly. Anticoagulation therapy was started postthromboembolectomy along with metoprolol XL 125 mg daily to resolve the sinus tachycardia.

Due to the recurrence of left knee pain, repeat incision and drainage were performed; cultures again showed few WBCs and no growth or crystals.

By May 19, the patient had clinical improvement; blood cultures remained negative. The patient remained on cefazolin via a peripherally inserted central catheter.

The patient was discharged to a long‐term, acute care facility for continued IV cefazolin and wound care management. The patient was afebrile, stable, and ambulatory. Follow‐up appointments for this patient were scheduled with infectious disease and orthopedic surgery.

## 3. Discussion

*S. aureus* is a major culprit in bacteremia and is often associated with high mortality and morbidity. This case exemplifies the aggressive potential of MSSA to disseminate hematogenously and result in multisystemic infection involving bone, muscle, soft tissue, and intravascular thrombi.

One of the most significant features of this case was the extensive spinal epidural abscess extending from T2 to T6, causing central canal stenosis and spinal cord compression. Spinal epidural abscesses are rare, with an incidence of 0.2 to 2.8 cases per 10,000 annually [15]. However, immunocompromised states and spinal surgery increase the propensity for an individual to develop a spinal epidural abscess [15]. The patient had a prior medical history that was significant for both conditions. The most common causative antimicrobial agent of spinal epidural abscesses is *S. aureus* [16, 17]. The T6 laminectomy and evacuation of the epidural abscess are consistent with a similar case of disseminated MSSA bacteremia that led to a spinal epidural abscess [18].

In addition to spinal involvement, the patient developed multiple abscesses spanning across the axial and appendicular skeletons. This is a typical well‐documented complication due to *S. aureus*’ ability to disseminate and cause deep‐seated abscesses in virtually every organ tissue [19]. Another known complication of metastasis of *S. aureus* is the involvement of multiple joints, including the sternoclavicular joint, spine, and knees [20], which also occurred in this case. The patient’s pseudogout was likely precipitated by the systemic inflammatory stress from the disseminated MSSA into joint spaces, along with transient hypocalcemia and recent corticosteroid exposure. Multiple abscesses and joint infections across anatomical compartments emphasize the high virulence of MSSA bacteremia and its capacity to spread.

Another complexity was identifying a large multilobular right atrial mass on TEE. Although there was an initial suspicion of infective endocarditis, which is commonly seen in MSSA bacteremia [21], the imaging and culture results suggested a bland thrombus. Evaluation by hematology did not reveal an inherited thrombophilia in this patient. However, family history was significant for multiple first‐degree relatives with instances of deep vein thromboses and clotting disorders, suggesting a possible predisposition. The percutaneous suction thromboembolectomy was a critical intervention to remove this potential embolic source. This case reinforces the diagnostic overlap between infective endocarditis and intracardiac thrombi in bacteremia patients with ongoing embolic risk, necessitating a nuanced diagnostic approach and considerations for advanced imaging.

Current treatment guidelines for MSSA recommend beta‐lactam antibiotic therapy with nafcillin, oxacillin, or cefazolin [22]. Despite appropriate antibiotic therapy with cefazolin, the patient exhibited persistent and evolving inflammatory collections. These involve recurrent abscess drainages and joint effusions even as blood cultures have cleared. This suggests that in this case of disseminated MSSA, aggressive source control, including incision and drainage in addition to antimicrobial therapy, remains central to effective management.

## 4. Conclusion

This case illustrates disseminated *S. aureus* bacteremia’s aggressive and multifocal nature, even when caused by methicillin‐sensitive strains. Despite susceptibility to beta‐lactam antibiotics, MSSA demonstrated the potential to be widespread, resulting in spinal epidural abscess. While antimicrobial therapy remains the cornerstone of treatment, surgical and procedural interventions were critical in achieving infection clearance and functional recovery. Broad and timely imaging, early source control, aggressive intervention, and close interdisciplinary collaboration remain the key in improving outcomes in disseminated MSSA infections. Prevention of future instances of disseminated MSSA bacteremia may be accomplished through patient education on the aseptic injection technique and early evaluation of skin trauma.

## Funding

No funds were received.

## Consent

Informed written consent was obtained from the patient for publication of this manuscript, including images or other personal or clinical details of the patient.

## Conflicts of Interest

The authors declare no conflicts of interest.

## Data Availability

The data that support the findings of this study are available from the corresponding author upon reasonable request.
